# P-963. Development, Delivery, and Evaluation of the Texas Epidemic Public Health Institute (TEPHI) Infection Control Lecture Series

**DOI:** 10.1093/ofid/ofae631.1153

**Published:** 2025-01-29

**Authors:** Kayla Ruch, Anabel Rodriguez, Janelle Rios, Luis Ostrosky-Zeichner, Eric L Brown

**Affiliations:** Texas Epidemic Public Health Institute (TEPHI), Houston, TX, USA The University of Texas Health Science Center at Houston, School of Public Health, Houston, TX, USA, Houston, Texas; Texas A&M University, School of Public Health, College Station, TX, USA, College Station, Texas; The University of Texas Health Science Center at Houston, School of Public Health, Houston, TX, USA, Houston, Texas; McGovern Medical School. UTHealth, Texas, Texas; University of Texas School of Public Health, Houston, Texas

## Abstract

**Background:**

The Texas Epidemic Public Health Institute (TEPHI) is a state agency of higher education headquartered at UTHealth Houston School of Public Health. With a mission to keep Texans healthy and the economy strong by preparing for the next infectious disease outbreak, TEPHI’s Small Rural Healthcare Preparedness working group developed, delivered, and evaluated a pilot infection prevention and control webinar series called *Infection Control* for rural-serving health organizations.

**Table 1:**
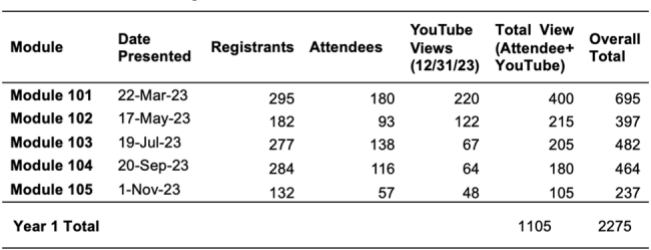
Modular Registration, Attendance, and YouTube Views

**Methods:**

Pilot data was collected from year one of the *Infection Control* series. Data sources included series attendee registration forms and attendance records (stored in WebEx Webinar Platform) and from post-lecture evaluation surveys using Qualtrics. These data were analyzed using Qualtrics software.

**Table 2:**
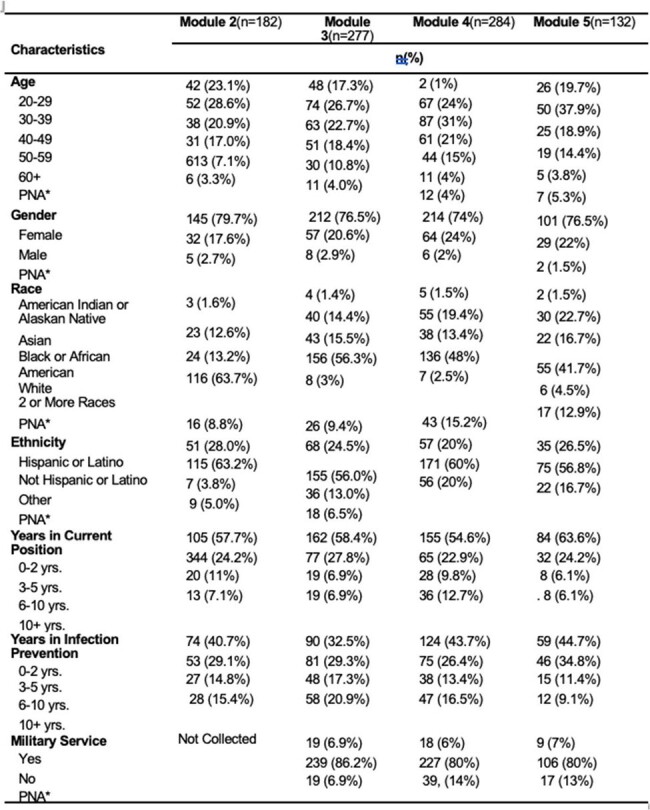
Infection Control series attendee demographic and occupational characteristics

**Results:**

A total of 1105 individuals attended or viewed the *Infection Control* series. Overall, response rate to the evaluation surveys, in general, was low. Even so, feedback was consistently very positive, noting a “high likelihood of future TEPHI infection prevention and control lecture attendance”. Additionally participant feedback was used to guide the active improvements for year two of the series.

**Table 3:**
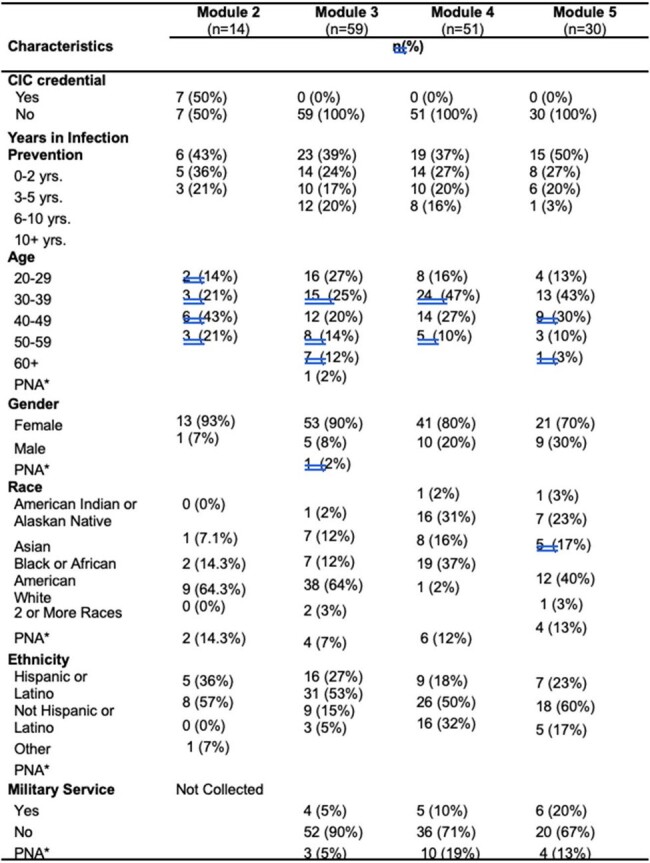
Infection Preventionist Module Registration Demographics

**Conclusion:**

*Infection Control* series attendees obtained a deeper understanding of relevant policies, procedures, components, and practices. By offering essential, accessible education on infection prevention and control at no cost to healthcare systems, administrators and healthcare providers working in these systems have gained the knowledge necessary to establish and sustain a safe environment for patients and staff in healthcare settings.

**Table 4:**
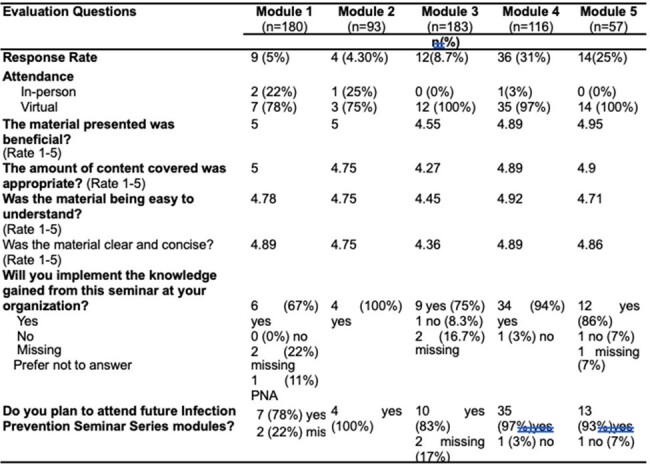
Modular Post-Survey Evaluations

**Disclosures:**

**Luis Ostrosky-Zeichner, MD, FACP, FIDSA, FSHEA, FECMM, CMQ**, Cidara: Advisor/Consultant|Enanta: Advisor/Consultant|F2G: Advisor/Consultant|Gilead: Advisor/Consultant|GSK: Advisor/Consultant|Melinta: Advisor/Consultant|Octapharma: Advisor/Consultant|Pfizer: Advisor/Consultant|Pfizer: Grant/Research Support|Pulmocide: Grant/Research Support|Scynexis: Grant/Research Support|Viracor: Advisor/Consultant

